# Supplementary material for: A comprehensive guide to CAN IDS data and introduction of the ROAD dataset
Source: PLoS One. 2024 Jan 22;19(1):e0296879. doi: 10.1371/journal.pone.0296879 (PMC10802965; doi:10.1371/journal.pone.0296879)
Supplement: Attachment — Submitted filename: response to reviewers.pdf [file pone.0296879.s002.pdf]

Second Round of Reviews

Response to the Reviewer #1: We appreciate the reviewers' time and the detail of the comments. Through the detailed reviews we received, we have significantly improved the quality of our paper, so our sincere thanks.

Response to Reviewer #2: We appreciate the reviewer's comments. We believe that our responses will clarify all concerns raised by the reviewer. We break down the reviewer's concerns into specific points (shown in italics) and then follow with our response. Changes in the manuscript have been highlighted in red.

1. *The article is weak in analysis and has not yet been improved despite being directed extensively by comments. No significant contribution was highlighted. It also lacks references that shed light on a broad scope of the problem. Some references have been suggested that researchers have not addressed.*

   We addressed all of reviewer's comments from the first round of review. We are attaching here our previous responses for your reference. We believe that after addressing reviewer's comments, our article certainly improved.

   We added more discussion to highlight the contribution of our work. Specifically, we added the following in the contributions subsection (Section 1.4): "The ROAD dataset fills gaps in the available CAN IDS datasets. The fabrication attacks isolate a targeted ID and send a single frame just after the ambient frame (see Section 4.1.2). This is the most stealthy fabrication attack possible and is not present in of the fabrication attacks of other datasets. This allows the most thorough testing of detectors of fabrication attacks (e.g. timing-based or AID-sequence-based detectors). Secondly, by omit- ting only the targeted IDs from these attacks we simulate a masquerade attack, which is not present in other datasets. Thirdly, we include advanced attacks which is unique only to ROAD. These latter two attack types allow ROAD to uniquely test payload-based detectors seeking more sophisticated attacks than fabrication attacks. By design the ROAD dataset's attacks increase in difficulty to detect, which is not present in previous datasets."

   Lastly, with respect to suggested references, we appreciate you pointing us to these papers. After carefully consideration, we notice that they are all focused on secure messaging in IoT or cloud infrastructures. Therefore, we consider that they are not relevant to the current work focused on CAN datasets. Nevertheless, we have provided more recent references to the aforementioned attacks which were included in Section 1.1. In particular, we added the following references:

Jeong, W., Choi, E., Song, H., Cho, M., & Choi, J. W. (2022). Adaptive Controller Area Network Intrusion Detection System Considering Temperature Variations. IEEE Transactions on Information Forensics and Security, 17, 3925-3933.

Zhao, Q., Chen, M., Gu, Z., Luan, S., Zeng, H., & Chakrabory, S. (2022). CAN bus intrusion detection based on auxiliary classifier GAN and out-of-distribution detection. ACM Transactions on Embedded Computing Systems (TECS), 21(4), 1-30.

Hanselmann, M., Strauss, T., Dormann, K., & Ulmer, H. (2020). CANet: An unsupervised intrusion detection system for high dimensional CAN bus data. Ieee Access, 8, 58194-58205.

de Faveri Tron, A., Longari, S., Carminati, M., Polino, M., & Zanero, S. (2022, November). Canflict: exploiting peripheral conflicts for data-link layer attacks on automotive networks. In Proceedings of the 2022 ACM SIGSAC Conference on Computer and Communications Security (pp. 711-723).

Nichelini, A., Pozzoli, C. A., Longari, S., Carminati, M., & Zanero, S. (2023). Canova: a hybrid intrusion detection framework based on automatic signal classification for can. Computers & Security, 128, 103166.

Tariq, S., Lee, S., & Woo, S. S. (2020, March). CANTransfer: Transfer learning based intrusion detection on a controller area network using convolutional LSTM network. In Proceedings of the 35th annual ACM symposium on applied computing (pp. 1048-1055).

Moulahi, T., Zidi, S., Alabdulatif, A., & Atiquzzaman, M. (2021). Comparative performance evaluation of intrusion detection based on machine learning in in-vehicle controller area network bus. IEEE Access, 9, 99595-99605.

Hossain, M. D., Inoue, H., Ochiai, H., Fall, D., & Kadobayashi, Y. (2020, July). Long short-term memory-based intrusion detection system for in-vehicle controller area network bus. In 2020 IEEE 44th Annual Computers, Software, and Applications Conference (COMPSAC) (pp. 10-17). IEEE.

Kalkan, S. C., & Sahingoz, O. K. (2020, July). In-vehicle intrusion detection system on controller area network with machine learning models. In 2020 11th International Conference on Computing, Communication and Networking Technologies (ICCCNT) (pp. 1-6). IEEE.

Olufowobi, H., Young, C., Zambreno, J., & Bloom, G. (2019). Saiducant: Specification-based automotive intrusion detection using controller area network (can) timing. IEEE Transactions on Vehicular Technology, 69(2), 1484-1494.

Moriano, P., Bridges, R. A., & Iannacone, M. D. (2022). Detecting CAN Masquerade Attacks with Signal Clustering Similarity. In: Workshop on Automotive and Autonomous Vehicle Security (AutoSec); 2022. p. 1-8

Shahriar, Md Hasan and Xiao, Yang and Moriano, Pablo and Lou, Wenjing & Hou, Y. Thomas (2023). CANShield: Deep Learning-Based Intrusion Detection Framework for Controller Area Networks at the Signal-Level. IEEE Internet of Things Journal.

We would like to thank Reviewer #2 again for taking the time to review our manuscript.

Response to the Reviewer #1: We appreciate the reviewer's comments. We include each of the reviewer's original comments in breakdown points in italics and then follow with our response. Changes in the manuscript have been highlighted in red. We believe that our responses will clarify all concerns raised by the reviewer.

1. *The introduction explains well the work environment and the proposed method, but it lacks the conclusions reached by the researcher based on specific criteria. In addition, the line alignment is not formatted.*

   Thank you for the suggestion. We have added a concluding statement in the introduction detailing our contributions. We also double checked that we have the latex template from the journal's submission site. The line formatting is as provided.

   Action: We have added the following to the introduction, specifically in the contributions subsection (Section 1.4): "The ROAD dataset fills gaps in the available CAN IDS datasets. The fabrication attacks isolate a targeted ID and send a single frame just after the ambient frame (see Section 4.1.2). This is the most stealthy fabrication attack possible and is not present in of the fabrication attacks of other datasets. This allows the most thorough testing of detectors of fabrication attacks (e.g. timing-based or AID-sequence-based detectors). Secondly, by omitting only the targeted IDs from these attacks we simulate a masquerade attack, which is not present in other datasets. Thirdly, we include advanced attacks which is unique only to ROAD. These latter two attack types allow ROAD to uniquely test payload-based detectors seeking more sophisticated attacks than fabrication attacks. By design the ROAD dataset's attacks increase in difficulty to detect, which is not present in previous datasets."

2. *Many types of CAN attacks are not referenced. Such as [f]abrication [a]ttacks and [s]uspension [a]ttacks. Please pay attention to adding references to the paragraphs extracted from their original references.*

   Thank you for your comment. Our three categories of attacks (Fabrication, Suspension, Masquerade) follows Cho & Shin 2016 [1]. In addition, we confirm that we add relevant references to paragraphs extracted from their original references. Please see Section 2.1.1.

   Action: We have now cited this in each of the three attack category subsections. In particular, Section 2.2.2 reads now: "An example from the literature is provided by Cho & Shin [1]'s bus off attack, where error counting is manipulated until an ECU is disallowed from speaking. After this bus off attack, the ECU will not transmit its messages; hence, those AIDs would be suspended."

3. *In [p]revious [d]atasets, it would have been better to shorten much of the narrative by adding tables that express the differences between the types of [d]atasets according to the classifications mentioned. Thus, we provide easy follow-up and comparison.*

   Thank you for your comment. We have provided three tables comparing, contrasting, and overall presenting previous datasets and ours, which include references, website, year, information about the data provided (real/synthetic, raw CAN, signal-translated, number of vehicles, total time) and many fields describing the attacks in each data (types of fabrication, synthetic, masquerade, other, and whether they are timing opaque). Please see Tables 1-3. After looking again

at our descriptions of the previous datasets, we are uncertain what other information could be usefully put into a table. Finally, we believe the discussion in each dataset's description is worthwhile and needed.

4. *The scientific term is mentioned with its abbreviation at the first appearance only.*

   We appreciate the reviewer's comment on this issue. We have reviewed the paper again and removed any occurrences of introducing acronyms after the first appearance of the acronym.

   Action: We have update the paper and removed any occurance of a repeat introduction to a scientific term with abbreviations. We ensure the reviewer that the scientific terms have been introduced once. In particular, the following acronyms were used and point of introduction was recorded:

   "ROAD"- Introduced in the Abstract as "we present the Real ORNL Automotive Dynamometer (ROAD)"

   "ECU" - Introduced in Introduction as "Electronic Control Units (ECUs)"

   "CAN" - Introduced in Abstract as "Controller Area Networks (CANs)"

   "IDS" - Introduced in the Abstract as "we introduce the first comprehensive guide to the existing open CAN intrusion detection system (IDS) datasets"

   "OBD-II" - Introduced in Introduction Section as "mandatory on-board diagnostics II (OBD-II) ports"

   "ML" - Introduced in the Introduction Section as "Machine-Learning-Based (ML-based) methods"

5. *The researcher did not present a comparison between ROAD Dataset and other previous types.*

   Thank you for your comment. We have provided tables comparing existing datasets. Please refer to Tables 1-3 and our response to your concern #3. In addition, we have added key features on how our proposed dataset compares to other datasets in the CAN IDS space.

   Action: We have added the following to the introduction, specifically in the contributions subsection (Section 1.4): "The ROAD dataset fills gaps in the available CAN IDS datasets. The fabrication attacks isolate a targeted ID and send a single frame just after the ambient frame (see Section 4.1.2. This is the most stealthy fabrication attack possible and is not present in of the fabrication attacks of other datasets. This allows the most thorough testing of detectors of fabrication attacks (e.g. timing-based or AID-sequence-based detectors). Secondly, by omitting only the targeted IDs from these attacks we simulate a masquerade attack, which is not present in other datasets. Thirdly, we include advanced attacks which is unique only to ROAD. These latter two attack types allow ROAD to uniquely test payload-based detectors seeking more sophisticated attacks than fabrication attacks. By design the ROAD dataset's attacks increase in difficulty to detect, which is not present in previous datasets."

6. *Organizing references according to a unified format.*

   Thank you for the suggestion. We have reread the paper and updated the references.

   Action: We updated all citations throughout the entire paper and compiled them according to the journal's standard.

We would like to thank Reviewer #1 again for taking the time to review our manuscript.

First Round of Reviews

Response to Reviewer #2: We appreciate the reviewer's comments. We believe that our responses will clarify all concerns raised by the reviewer. We break down the reviewer's concerns into specific points (shown in italics) and then follow with our response. Changes in the manuscript have been highlighted in red.

1. *In abstract, the criteria used to present a dataset and the types of attacks must be mentioned and the types of attacks must be mentioned.*

   Thank you for the suggestion. We have included details about the types of attacks contained in the ROAD dataset in the abstract.

   Action: We have added the following to the abstract: "We categorize attacks on CANs including fabrication (adding frames, e.g., flooding or targeting and ID), suspension (removing an ID's frames), and masquerade attacks (spoofed frames sent in lieu of suspended ones). We provide a quality analysis of each dataset; an enumeration of each datasets' attacks, benefits, and drawbacks; categorization as real vs. simulated CAN data and real vs. simulated attacks; whether the data is raw CAN data or signal-translated; number of vehicles/CANs; quantity in terms of time; and finally a suggested use case of each dataset."

2. *On page 2, Introduction section, "As a result, proposed detection techniques are often not tested on appropriate data due to lack of availability.", What is the evidence for this claim?*

   Thanks for the comment. We have provided evidence for this claim.

   Action: We have added the following clarifying example to the introduction: "E.g., Hossain et al. [2] simulate attacks in real CAN data by adding frames in order to validate an LSTM-based CAN IDS. In theory their IDS may detect much more subtle attacks, e.g., masquerade attacks (see Attack Section) but without available data, this cannot and was not tested. Further, using simulated data or attacks limits fidelity compared to real vehicular CAN data with real, and physically verified, attacks."

3. *The types of attacks and their names must be mentioned in the Introduction Section.*

   Thanks for the suggestion. We have added a description of types of attacks in the introduction.

   Action: We added the following to the introduction: "More specifically, the dataset provides real Denial of Service (DoS), fuzzing, and stealthy targeted ID fabrication attacks, simulated suspension attacks, simulated masquerade attacks, and an advanced attack in which user controls cease to match vehicle behavior." and when discussing attacks (end of Section 1) we have added: "In particular, we partition attacks into categories fabrication attacks (which add frames to the bus, e.g., denial of service (DOS), fuzzing), suspension attacks (which prevent frames from being sent thereby removing them from the bus), masquerade attacks (replacing legitimate frames with malicious frames), and other. Specific definitions of types of these attacks are discussed."

4. *From line 55-70, lacks more modern references.*

   Thank you for the suggestion. We have reviewed several recent papers and included them to Section 1.1.

   Action: We updated Section 1.1 lines 55-65 to include the following references:

Jeong, W., Choi, E., Song, H., Cho, M., & Choi, J. W. (2022). Adaptive Controller Area Network Intrusion Detection System Considering Temperature Variations. IEEE Transactions on Information Forensics and Security, 17, 3925-3933.

Zhao, Q., Chen, M., Gu, Z., Luan, S., Zeng, H., & Chakrabory, S. (2022). CAN bus intrusion detection based on auxiliary classifier GAN and out-of-distribution detection. ACM Transactions on Embedded Computing Systems (TECS), 21(4), 1-30.

Hanselmann, M., Strauss, T., Dormann, K., & Ulmer, H. (2020). CANet: An unsupervised intrusion detection system for high dimensional CAN bus data. Ieee Access, 8, 58194-58205.

de Faveri Tron, A., Longari, S., Carminati, M., Polino, M., & Zanero, S. (2022, November). Canflict: exploiting peripheral conflicts for data-link layer attacks on automotive networks. In Proceedings of the 2022 ACM SIGSAC Conference on Computer and Communications Security (pp. 711-723).

Nichelini, A., Pozzoli, C. A., Longari, S., Carminati, M., & Zanero, S. (2023). Canova: a hybrid intrusion detection framework based on automatic signal classification for can. Computers & Security, 128, 103166.

Tariq, S., Lee, S., & Woo, S. S. (2020, March). CANTransfer: Transfer learning based intrusion detection on a controller area network using convolutional LSTM network. In Proceedings of the 35th annual ACM symposium on applied computing (pp. 1048-1055).

Moulahi, T., Zidi, S., Alabdulatif, A., & Atiquzzaman, M. (2021). Comparative performance evaluation of intrusion detection based on machine learning in in-vehicle controller area network bus. IEEE Access, 9, 99595-99605.

Hossain, M. D., Inoue, H., Ochiai, H., Fall, D., & Kadobayashi, Y. (2020, July). Long short-term memory-based intrusion detection system for in-vehicle controller area network bus. In 2020 IEEE 44th Annual Computers, Software, and Applications Conference (COMPSAC) (pp. 10-17). IEEE.

Kalkan, S. C., & Sahingoz, O. K. (2020, July). In-vehicle intrusion detection system on controller area network with machine learning models. In 2020 11th International Conference on Computing, Communication and Networking Technologies (ICCCNT) (pp. 1-6). IEEE.

Olufowobi, H., Young, C., Zambreno, J., & Bloom, G. (2019). Saiducant: Specification-based automotive intrusion detection using controller area network (can) timing. IEEE Transactions on Vehicular Technology, 69(2), 1484-1494.

Moriano, P., Bridges, R. A., & Iannacone, M. D. (2022). Detecting CAN Masquerade Attacks with Signal Clustering Similarity. In: Workshop on Automotive and Autonomous Vehicle Security (AutoSec); 2022. p. 1-8

Shahriar, Md Hasan and Xiao, Yang and Moriano, Pablo and Lou, Wenjing & Hou, Y. Thomas (2023). CANShield: Deep Learning-Based Intrusion Detection Framework for Controller Area Networks at the Signal-Level. IEEE Internet of Things Journal.

5. *In line 91, "40 CAN IDS papers surveyed", these references must be cited.*

   Thank you for the suggestion. We understand that we were not clear with the previous statement. We performed a literature search to discover papers that are based on CAN IDS methods and recorded the trends in the bar plots. See Fig. 1.

   Action: The following was added to the introduction in Section 1.1:

   "A quick meta-analysis demonstrates the recent trends (up to 2023) of CAN IDS papers and shows the current interest in the topic. Fig. 1 highlights these trends based on yearly publications and category breakdown, showcasing a steady yearly trend of CAN IDS publications since 2014."

6. *Please add surveys for recent years.*

   Thank you for the suggestion. We have identified the following survey published in 2023 based on CAN IDS:

   Rajapaksha, S., Kalutarage, H., Al-Kadri, M. O., Petrovski, A., Madzudzo, G., & Cheah, M. (2023). Ai-based intrusion detection systems for in-vehicle networks: A survey. ACM Computing Surveys, 55(11), 1-40.

   Action: The following was added to the Introduction in Section 1.1:

   "In [3], Rajapaksha, et al. review and categorize recent AI-based IDSs for securing In-Vehicle Networks (IVNs), with a particular focus on the CAN bus, offering valuable insights into effective AI algorithms and their applications in the context of IVN cybersecurity. The study highlights the superior detection capabilities of deep learning algorithms, particularly LSTM and GRU-based ensemble models, in the context of IVN security, along with promising trends in unsupervised learning, transfer learning, and the potential to combine physical characteristics with CAN data frame features for enhanced attack detection."

7. *The propriety CAN signal problem must be added to the article, even if the addition is in brief with tables.*

   Thank you for the suggestion. We have now discussed the CAN signal problem and included a Table for comparison purposes.

   Action: We have added a detailed description of the proprietary CAN signal problem and a table comparing previous reverse engineering works as suggested. Specifically, we added the following subsection (Sec 1.1.1 CAN Reverse Engineering Problem) and a table (see Table 4). In particular, we added:

   "The asymmetric growth in the field—in particular the disproportionate number of publications on methods that are timing/frequency-based (and to a lesser extent payload-based) as compared to signal-based—is a direct result of the proprietary CAN signal encodings issue. Original equipment manufacturers (OEMs, e.g., Subaru, Ford) of passenger vehicles hold secret their proprietary encodings of signals in the CAN data fields and vary the encodings across models. Consequently, though researchers can easily add a node to monitor and send CAN

	Tokenization	Endianness	Encoding	Interpretation
Jaynes et al. (2016) [11]	○	○	○	◐
Markowitz & Wool (2017) [9]	●	○	○	○
Huybrechts et al. (2018) [12]	◐	○	○	◐
Nolan et al.'s TANG (2018) [8]	●	○	○	○
Marchetti & Stabili's READ (2018) [7]	●	○	○	○
Verma et al.'s ACTT (2018) [6]	●	○	○	●
Pesé et al. LibreCAN (2019) [5]	●	○	○	●
Young et al. (2020) [13]	○	○	○	◐
Buscemi et al. (2021) CANMatch [14]	●	●	○	●
Verma et al. (2021) CAN-D [4]	●	●	●	●

messages on most vehicles, the data is not understandable. Thus, most have focused on methods that do not require knowledge of signal encodings. Indeed a few researchers have paired with OEMs or done some manual reverse engineering to obtain and develop IDSs based on the decoded CAN signals, though these developments are not vehicle-agnostic.

Although the proprietary CAN signal problem is not addressed in this paper, it is necessary context for the issue of availability of real automotive CAN data with advanced attacks present. Partially reverse engineered signal mappings are emerging online, and a small subfield has emerged with the goal of automating the reverse engineering of signals from automotive CAN data [4, 5, 6, 7, 8, 9]). Verma et al. [4] provide a comprehensive treatment of the problem as well as a survey of the previous work. In particular, the signal-reverse engineering problem can be broken into four sub-problems: (1) tokenization - identifying the signal boundaries within the data field (e.g., in a 64-bit data field, bits 1-8 may encode wheel speed, bit 9 a binary indicator of cruise control, bit 10 unused, bits 12-16 a temperature signal, ...); (2) endianness - for signals crossing a byte boundary the order of the bytes is needed; (3) integer-to-binary encoding - usually base 2 or 2's complement encoding is used; (4) interpretation - an affine mapping of each signal to achieve the correct units and labeling the signal with what it communicates (e.g., speed in mph). Table 4 itemizes previous CAN reverse engineering works and the portions of the four sub-problems to which they contribute. For a comprehensive survey on CAN reverse engineering, we refer the reader to prior work by Buscemi et al. [10] for further details. Overall, signal reverse engineering can facilitate CAN IDS research and, more generally, may enable a wide variety of downstream automotive technologies, e.g., OpenPilot aftermaket driver assistance technology (`https://comma.ai/`)."

8. *On page 5, Section "Problem Addressed". Does the current CAN IDS fail to prevent attacks in the real world? Examples should be mentioned.*

We are not aware of attacks "in the wild", that is, that have been proven to be used against actual drivers. Although numerous CAN IDS technologies have been proposed in research, our efforts to locate a real-world implementation of such a tool have been unsuccessful. It should be noted that IDSs act as a reporting and alerting tool, and do not inherently prevent attacks or their effects. To that end,

the exploration of Intrusion Prevention Systems (IPSs) is an emerging area of research for CAN-based vehicles. To our knowledge, these devices have also not been deployed in real-world environments. The ROAD dataset aims to improve the efficacy of this research and deployment of security technologies by providing an open research dataset applicable to testing and improving IDS.

9. *On page 6, line 189-194, what is the difference from Reference 28, is that its data is considered standard.*

   Thanks for your comment. In our work, we do not propose a new taxonomy of CAN IDSs as done by Ref. [28]. Instead, we echo that given the lack of high-quality and open datasets, it is a challenging to compare different CAN IDS approaches. This hinders progress in the area. Our work and released dataset contributes to close this gap by allowing more transparent comparison of IDS approaches.

10. *In contributions, you must briefly mention the environment in which the data was trained for three hours.*

    Thanks for the suggestion. We have added details on the data collection environment.

    Action: We have added the following to contributions in Section 4: "The dataset provides ample (3 hours) training data with no attacks collected when driving on actual roads (not dynamometer)."

11. *On page 9, 2.1 Can Protocol, An example must be given to illustrate the transfer of messages between ECUs, indicating the fields of payload.*

    Thanks for the suggestion. Our text includes the following example in Section 2.1: "For example, the PCM may transmit: ID `0x102` with data field containing engine RPM, vehicle speed, and odometer signals every 0.05s, and ID `0x45D` with data field containing signals encoding the angle of the gas and brake pedals every 0.01s." Further, we have included an image depicting the different fields of a CAN data frame (see Fig. 2).

12. *Recent references to the aforementioned attacks must be cited, such as: https://ieeexplore.ieee.org/abstract/document/9274321, https://www.mdpi.com/2079-9292/12/17/3688, https://www.mdpi.com/2224-2708/11/3/55*

    Thank you for the suggestion. While we appreciate you pointing us to these papers, they are all focused on secure messaging in IoT or cloud infrastructures. They are not relevant to the current work. Nevertheless, we have provided more recent references to the aforementioned attacks. Please refer to the details in our answer to your previous comment #4.

13. *On page 10, the difference between DOS and Fuzzy attacks must be clarified. Are there examples in the real world of these attacks?*

    On page 10, the DOS Attack definition says (Section 2.2.1): "Messages with ID 0x000 and an arbitrary payload are injected at a high frequency..." while the Fuzzing Attack definition says: "Messages with random IDs and arbitrary payloads are injected at a high frequency. The bus becomes occupied with mostly injected messages, displacing real messages, and resulting in similar behavior to the DoS attack. Unlike the DoS attack, injected messages may have an ID that appears in normal traffic, so receiver nodes expecting these ID messages will read and use the information in the malicious payload, causing a wide variety of

unexpected results. To illustrate these effects we reference a video of a fuzzing attack[1]." We believe this clearly articulates the difference between the two. We are unaware of such attacks in the wild, but we have provided a video of such an attack on a real vehicle.

14. *On page 12, the mechanism of the suspension attack is unclear.*

    An example from the literature is provided by Cho & Shin 2016, the bus off attack, where error counting is manipulated until an ECU is disallowed from speaking. After this bus off attack, the ECU will not transmit its messages; hence, those AIDs would be suspended. We hope this clarifies the mechanism of the suspension attack.

15. *Section 3 [p]revious [d]atasets, [u]sage area must be added for each attack.*

    Thank you for the suggestion. We have discussed usage in previous datasets.

    Action: For all datasets, we have now provided usage information, if available, in the publication of the dataset. Please refer to Section 3 for details on each of the dataset when applicable.

16. *Section 4. Introducing the ROAD [d]ataset, at least mention the name of the vehicle manufacturer, but not its model, according to your organization's regulations.*

    Thank you for your comment. Our organization's lawyers will not allow this.

17. *On page 20, why was 0.005 seconds chosen?*

    It was chosen so that the attack would be relatively easy to detect–this should be the easiest attack in the ROAD dataset to detect.

    Action: We have stated this in the paper. Please see Section 4.1.1.

18. *A table must be added in the results section showing the difference between the presented database and its predecessors.*

    We feel this is best communicated via prose than another table. Please note that we have three tables including all previous datasets and our new dataset. Please see Tables 1-3. These tables allow direct comparison of the datasets' characteristics and the attacks they present. As for placement of the tables, we have moved them to early in the introduction so that the takeaways most useful to other researchers (e.g., links to datasets, and summary of their contents) are up front.

    Action: We have added the following to the introduction, specifically in the contributions subsection (Section 1.4): "The ROAD dataset fills gaps in the available CAN IDS datasets. The fabrication attacks isolate a targeted ID and send a single frame just after the ambient frame (see Section 4.1.2). This is the most stealthy fabrication attack possible and is not present in of the fabrication attacks of other datasets. This allows the most thorough testing of detectors of fabrication attacks (e.g. timing-based or AID-sequence-based detectors). Secondly, by omitting only the targeted IDs from these attacks we simulate a masquerade attack, which is not present in other datasets. Thirdly, we include advanced attacks which is unique only to ROAD. These latter two attack types allow ROAD to uniquely test payload-based detectors seeking more sophisticated attacks than fabrication attacks. By design the ROAD dataset's attacks increase in difficulty to detect, which is not present in previous datasets."

[1] https://youtu.be/80A5IhvwsJU

We would like to thank Reviewer #2 again for taking the time to review our manuscript.

References

[1] Cho and Shin, "Error handling of in-vehicle networks makes them vulnerable," in *SIGSAC*, ACM, 2016.

[2] M. D. Hossain, H. Inoue, H. Ochiai, D. Fall, and Y. Kadobayashi, "Lstm-based intrusion detection system for in-vehicle can bus communications," *IEEE Access*, vol. 8, pp. 185489–185502, 2020.

[3] S. Rajapaksha, H. Kalutarage, M. O. Al-Kadri, A. Petrovski, G. Madzudzo, and M. Cheah, "Ai-based intrusion detection systems for in-vehicle networks: A survey," *ACM Computing Surveys*, vol. 55, no. 11, pp. 1–40, 2023.

[4] M. E. Verma, R. A. Bridges, J. J. Sosnowski, S. C. Hollifield, and M. D. Iannacone, "CAN-D: A modular four-step pipeline for comprehensively decoding controller area network data," *IEEE TVT*, vol. 70, no. 10, pp. 9685–9700, 2021.

[5] M. D. Pesé, T. Stacer, C. A. Campos, E. Newberry, D. Chen, and K. G. Shin, "LibreCAN: Automated CAN message translator," in *SIGSAC CCS*, ACM, 2019.

[6] M. Verma, R. Bridges, and S. Hollifield, "ACTT: Automotive CAN Tokenization & Translation," in *CSCI*, IEEE, 2018.

[7] Marchetti and Stabili, "READ: Reverse engineering of automotive data frames," *IEEE Transactions on Info. Foren. & Sec.*, vol. 14, no. 4, pp. 1083–1097, 2018.

[8] B. C. Nolan, S. Graham, B. Mullins, and C. S. Kabban, "Unsupervised time series extraction from controller area network payloads," in *(VTC-Fall)*, IEEE, 2018.

[9] Markovitz and Wool, "Field classification, modeling and anomaly detection in unknown CAN bus networks," *Vehicular Communications*, vol. 9, 2017.

[10] A. Buscemi, I. Turcanu, G. Castignani, A. Panchenko, T. Engel, and K. G. Shin, "A survey on controller area network reverse engineering," *IEEE Communications Surveys & Tutorials*, 2023.

[11] M. Jaynes, R. Dantu, R. Varriale, and N. Evans, "Automating ecu identification for vehicle security," in *ICMLA*, 2016.

[12] T. Huybrechts, Y. Vanommeslaeghe, D. Blontrock, G. Van Barel, and P. Hellinckx, "Automatic reverse engineering of can bus data using machine learning techniques," in *Advances on P2P, Parallel, Grid, Cloud and Internet Computing: Proceedings of the 12th International Conference on P2P, Parallel, Grid, Cloud and Internet Computing (3PGCIC-2017)*, pp. 751–761, Springer, 2018.

[13] C. Young, J. Svoboda, and J. Zambreno, "Towards reverse engineering controller area network messages using machine learning," in *2020 IEEE 6th World Forum on Internet of Things (WF-IoT)*, pp. 1–6, IEEE, 2020.

[14] A. Buscemi, I. Turcanu, G. Castignani, R. Crunelle, and T. Engel, "Canmatch: a fully automated tool for can bus reverse engineering based on frame matching," *IEEE Transactions on Vehicular Technology*, vol. 70, no. 12, pp. 12358–12373, 2021.
